# Field assessment of brix and firmness affecting *Drosophila suzukii* (Diptera: Drosophilidae) infestation in California sweet cherry cultivars

**DOI:** 10.1093/jee/toaf367

**Published:** 2026-01-24

**Authors:** Nicolas Buck, Brian E Gress, Frank G Zalom

**Affiliations:** Department of Entomology and Nematology, University of California, Davis, Davis, CA, United States; California Department of Food and Agriculture, Sacramento, CA, United States; Department of Entomology and Nematology, University of California, Davis, Davis, CA, United States

**Keywords:** spotted-wing drosophila, preference, *Prunus avium*

## Abstract

Spotted-wing Drosophila, *Drosophila suzukii* Matsumura (Diptera: Drosophilidae), is a damaging insect pest of sweet cherry fruit worldwide including in the central valley of California where it was first reported as an economic pest in spring 2009. The aim of this field-based study was to assess the relationship of Brix level and skin firmness on *D. suzukii* oviposition and infestation in 4 commercially important sweet cherry cultivars: Bing, Black Tartarian, Brooks, and Rainier. Results of this field study found that both higher Brix values and lower skin firmness resulted in increased fruit infestation in all varieties, highlighting the importance of these factors in host susceptibility. Other potential factors are also discussed as they relate to *D. suzukii* fruit infestation. Implications of these findings are discussed as well as how they might be used in future *D. suzukii* management in sweet cherries.

## Introduction

*Drosophila suzukii* Matsumura (Diptera: Drosophilidae), is one of the most damaging insect pests to the global soft and stone fruit industry (Kinjo et al. 2014). Native to Southeast Asia, the first records of *D. suzukii* in the United States and Europe were in California and Spain, respectively, in 2008 (Hauser 2011, Calabria et al. 2012, Atallah et al. 2014). Adult *D. suzukii* females damage fruit using their sharply serrated ovipositors to cut into ripe fruit and lay eggs, in contrast with the preference for dropped, rotten or decayed fruit observed amongst other Drosophilids (Atallah et al. 2014, Schetelig et al. 2018). The opening of the fruit by oviposition provides a pathway for pathogens to enter the fruit, further degrading it, and can also lead to greater risk of infestation by other Drosophilid flies (Walsh et al. 2011, Hamby et al. 2012, Cini et al. 2014, Asplen et al. 2015). *D. suzukii* are able to oviposit on fruit of a wide variety of hosts (Walsh et al. 2011, Kenis et al. 2016), with fruits of thinner exocarp exhibiting greater susceptibility due to ease of exocarp penetration (Bellamy et al. 2013), highlighting the risk this pest poses to soft and stone fruit growers.

Cherry was the eighth most valuable fruit and nut crop globally in 2010 (Elser et al. 2016). Worldwide sweet cherry production was 2.6 million tons in 2020 with Turkey the primary producer and the United States second (Hu et al. 2021, Reim et al. 2023, Chezanoglou et al. 2024). Over 95% of US sweet cherry production occurs in Washington, California, and Oregon (Bolda et al. 2010, Beers et al. 2011, Elser et al. 2016). Damage to blackberries, raspberries and cherries without appropriate management of *D. suzukii* has been estimated at $860 million in those 3 states (Walsh et al. 2011, Hamby et al. 2012), however effective management practices have significantly reduced these losses (Farnsworth et al. 2017).

There are several commercially important cherry cultivars currently produced in California, among them Bing, Brooks, Rainier and Black Tartarian, and orchards are typically planted with 2 or more cultivars to facilitate cross-pollination. Of these 4 cultivars, Bing and Brooks are the more widely grown, while Black Tartarian is the least studied (Schuster 2012, Chockchaisawasdee et al. 2016). Bing has been the dominant cultivar for decades, however Brooks, which ripens earlier in the season, has become a favored cultivar amongst California growers to meet early season market demand (Crisosto et al. 2002, Hu et al. 2021). Both Bing and Rainier are popular due to their large size, greater weight, higher Brix (sugar content) values, exocarp firmness and overall quality compared to other cultivars (Dangi et al. 2024). These 4 sweet cherry cultivars, while popular choices amongst growers along the west coast of the United States, also present a high risk for *D. suzukii* damage due to some of these desirable characteristics. However, cultivars such as Black Tartarian and Brooks ripen earlier than Bing and Rainier, and so may face lower *D. suzukii* pressure during their harvest period.

The vast majority of commercial sweet cherry production in California is carried out in the Central Valley, along with a variety of other stone fruits as well as other cultivated fruit crops such as blueberries, citrus, grapes, and raspberries. The widespread availability of suitable hosts and relatively extended period of fruit production makes management of *D. suzukii* damage a challenge (Haviland et al. 2016). Adult female *D. suzukii* use a mix of both chemosensation and mechanosensation to detect suitable hosts for oviposition, incorporating a range of factors (Karageorgi et al. 2017). In addition, host preference has been shown to vary with fruit ripeness and associated factors such as pH, firmness and Brix playing a role in host selection (Lee et al. 2011, Burrack et al. 2013, Stewart et al. 2014, Hamby et al. 2016). In Mexico, overripe or softer guavas exhibited higher infestation rates than guavas that had firmer exocarps (Lasa et al. 2017). Other research shows fruit quality metrics that relate to ripeness can influence host selection for oviposition (Lee et al. 2011). In grapes, early ripening varieties and those with thicker exocarp experienced significantly lower infestation rates and lower egg to adult development success than late season cultivars with thinner exocarp thickness, indicating the importance of exocarp condition, ripeness stage, and how it can influence the ability for *D. suzukii* to oviposit in fruit (Linder et al. 2014). Other studies show late-season cherry varieties with varying levels of exocarp firmness, Brix, and color also exhibited higher infestation rates than early-season cultivars that were tested, although this could be due to increased population densities exhibited later in the season (Lee et al. 2011, Papanastasiou et al. 2020).

To determine the relative susceptibility of 4 commercially important sweet cherry cultivars grown within mixed cultivar orchard blocks to *D. suzukii* infestation, we assessed if both Brix level and fruit firmness were significant factors in determining fruit infestation in a preliminary study on 5 cherry trees representing different varieties, then assessed infestation status by Brix level and exocarp firmness on the common sweet cherry cultivars Bing, Brooks, Black Tartarian (referred to as BT in graph legends) and Rainier in a subsequent 2-yr study. This study represents the first in-field trial in a natural setting that assesses the effects of Brix and firmness on sweet cherry infestation of 4 common commercially available cultivars over the fruit ripening period in contrast to laboratory-based studies on sweet cherry (Lee et al. 2011, Yang et al. 2024) and field-based studies on tart cherries (Kamiyama and Guédot 2019).

## Materials and Methods

Due to the difference in methodologies between the 2013 and 2017/2018 studies, the Materials and Methods section has been separated by study year, although both assessed the effects of Brix and firmness on sweet cherry infestation in the field, while the Results section has been separated by assessment type.

### 2013 Preliminary Study

The preliminary 2013 study aimed to identify significant effects of Brix and firmness on sweet cherry infestation in the field that would warrant a further, more developed study. This was carried out in a 10-tree sweet cherry teaching block at the UC Davis Pomology Farm (Yolo County, California, United States—38°32′21.1″N 121°47′36.4″W) from 5 May 2013 to 4 June 2013. The age of the trees was not known, but they had been planted with a mixture of unknown cultivars (labelled 1L, 1N, 2D, 2K, and 2M) over 25 yr earlier. It was not treated with insecticides or fungicides. Five of the trees were randomly selected to conduct the study. Every 3 to 5 d 100 ripe cherries were collected from each tree, placed into plastic ziplock bags, and returned to the Zalom lab at UC Davis where they were evaluated for infestation (determined in the 2013 study by the presence or absence of “stings”; dents in the cherry surface typically caused by *D. suzukii* oviposition, as well as the number of larvae and pupae per cherry). Of the 100 cherries, 40 were set aside individually in small plastic containers to allow for *D. suzukii* development. The remaining 60 cherries were tested for firmness (Firmtech 2, BioWorks, Inc, Cleveland, Ohio, United States). Of these 60, 10 randomly selected infested cherries and 10 uninfested fruit collected from the same tree on the same day were subjected to Brix measurement using an AO T/C Refractometer (Model 10430, American Optical Company, Vernon Hills, Illinois, United States).

### 2017 and 2018 Study

The 2017 and 2018 studies were conducted between 28 April – 5 June and 3 May – 31 May, respectively, at the UC Davis Plant Pathology Farm (Solano County, California, United States—38°31′20.5″N 121°45′54.5″W) in a mixed cultivar sweet cherry block consisting of 116 trees that were approximately 30 yr old. The block was not treated with insecticides or fungicides. Four cultivars, Bing, Black Tartarian, Brooks, and Rainier were selected for fruit sampling because they had somewhat different ripening dates, reflecting their use as cross-pollinators in commercial orchards. The trees selected were intentionally in close proximity to one another and not on the borders of the block in an attempt to reduce potential *D. suzukii* distribution effects. Cultivar identification of each tree was confirmed by PCR sequencing conducted by the Foundation Plant Services unit at UC Davis. In 2017, 2 trees were sampled for Black Tartarian, 1 for Brooks, 4 for Bing, and 1 for Rainier. In 2018, 2 trees were sampled for Black Tartarian, 1 for Brooks, 2 for Bing, and 2 for Rainier. Study trees varied somewhat between years primarily based on having sufficient fruit present to enable fruit collection throughout the anticipated sampling period. Eighty fruits per tree were collected on each sampling date every 3 to 5 d. Fruit were randomly sampled by collecting equal numbers from the north, south, east, and west quadrants of each tree and from high to low in the canopy. A ladder was used to reach the high canopy.

Each fruit was initially assessed under a stereomicroscope in the laboratory for the presence or absence of *D. suzukii* stings and eggs, if either were present, this was classified as a 1 for infestation status, indicating the presence of infestation. If these were not present in the fruit, it was classified as a 0, indicating an absence of infestation. Of the 80 fruits collected from each tree, firmness was measured from a random subsample of 20 fruit using a TA XT Plus Texture Analyzer (Texture Technologies Corp., Hamilton, Massachusetts, United States). Brix measurements were also recorded in 2017 using the same refractometer as in 2013. Temperature data was collated via National Oceanic and Atmospheric Association (NOAA) for the specific coordinates of the site in 2017 and 2018. The maximum, minimum, and average temperatures were recorded for each of the 16 sampling days, 10 in 2017 and 6 in 2018.

### Statistical Analysis

Larvae and pupae per fruit were recorded only for 2013. These data were collated by averaging the number of larvae and the number of pupae recorded per individual fruit in a subsample. To analyze mean number of larvae and pupae in 2013, generalized linear models (GLM) (McCullagh and Nelder 1989) were built with either Brix or firmness as predictors and temperature as a covariant. These models had a Gaussian distribution, and the response variable was log-transformed for model convergence. Further, to analyze infestation status as a binomial response, in 2013, we built a separate GLM model with either Brix or firmness as predictors, infestation status as the response (infested vs non-infested), and tree ID as a covariant. This model contained a binomial distribution.

To examine infestation status, the presence or absence of infestation of each fruit was recorded as a binary response in 2017 and 2018, in addition to 2013. To assess the effects of Brix (2017 only) or firmness on infestation status for 2017 and 2018 a GLM was built with either Brix or firmness as predictors, temperature and cultivar as additional fixed effects, and an interaction between Brix or firmness and cultivar. These models contained a binomial distribution. This interaction provides information on how Brix or firmness affects infestation status by cultivar.

To analyze differences in Brix (2017 only) and firmness levels between varieties in 2017 and 2018, ANOVAs and Tukey’s HSD tests were used. Cultivar was used as the predictor and Brix or firmness as the response variables.

A GLM was also used to assess the differences in minimum, average, and maximum temperatures at the site location in 2017 and 2018, using the minimum, average, and maximum temperature for each sampling day as the response variable, Year as the predictor variable and a Gaussian distribution. Lastly, Pearson product-moment correlation tests were run to assess the correlation between average temperature and sampling date in both years. Significance was determined with a *P* value of less than 0.05. All statistics were carried out using R version 4.5.0 (R Core Team 2025).

## Results

### Temperature Trends

**Fig. 1.**
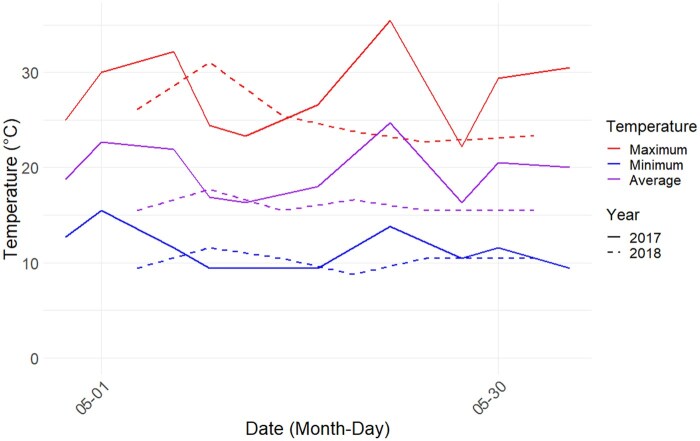
Maximum (top), minimum (bottom), and average (middle) temperatures for the period during which cherry fruit were collected in 2017 (smooth) and 2018 (dotted). Top, middle, and bottom temperature ranges include one smooth and one dotted line each.

We found that maximum (β = −2.379 ± 0.267, *t* = −8.899, *P* < 0.001), minimum (β = −1.145 ± 0.129, *t* = −8.82, *P* < 0.001), and average temperature (β = −3.507 ± 0.174, *t* = −20.14, *P* < 0.001) were all significantly higher in 2017 than 2018 (Fig. 1).

### Brix Assessments

**Fig. 2.**
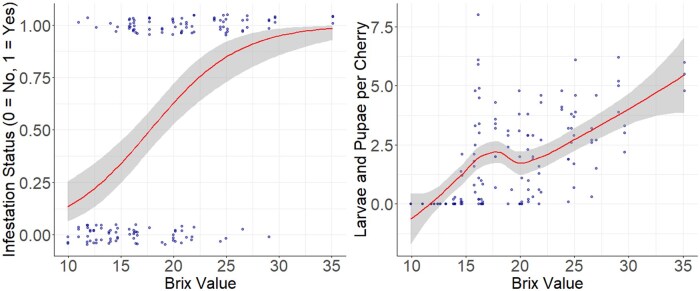
The relationship between fruit Brix value and probability of sweet cherry infestation status by *D. suzukii* (left, *P* < 0.001) and the number of larvae and pupae per cherry (right, *P* < 0.001) in the 2013 preliminary study. In both graphs, the smooth line represents the regression line, the band surrounding the smooth line represents the 95% CIs, and the dots represent data points. Some dots are overlapping and incorporated into the same dot, so not all datapoints are visible.

In the 2013 preliminary study, to examine the effects of Brix on fruit infestation status, we found that Brix value had a significant positive effect on the probability of fruit infestation status (β = 0.343 ± 0.059, *t* = −5.734, *P* < 0.001, Fig. 2) and on larvae and pupae per cherry (β = 0.078 ± 0.008, *t* = 9.5, *P* < 0.001, Fig. 2).

**Fig. 3.**
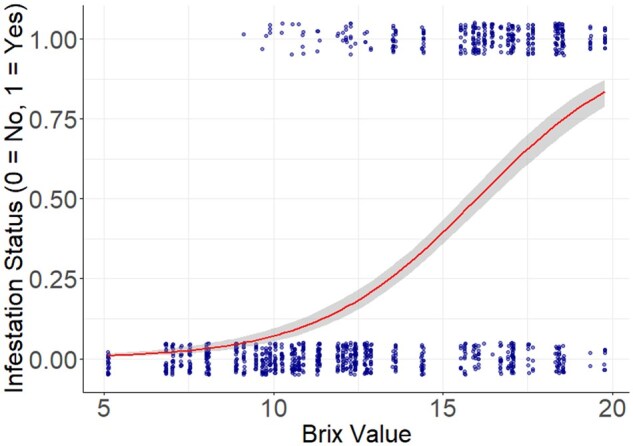
Relationship between Brix value and the probability of sweet cherry fruit being infested by *D. suzukii* in 2017 (*P* < 0.001, all cultivars included). The smooth regression line represents the probability of infestation, the band surrounding the smooth line represents the 95% CIs, and the dots represent data points. Some dots are overlapping and incorporated into the same dot, so not all datapoints are visible.

When assessing the effects of Brix on infestation status in 2017, we found Brix value had a positive, significant effect on cherry infestation status, showing an increase in infestation with an increase in Brix value (β = 0.428 ± 0.038, *t* = 11.262, *P* < 0.001, Fig. 3).

**Fig. 4.**
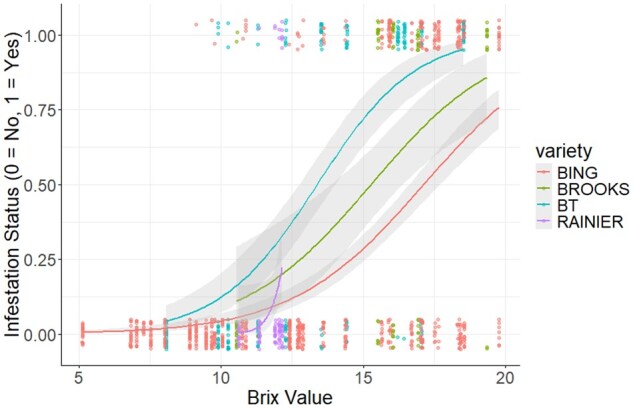
Relationship between Brix value and the probability of each sweet cherry cultivar being infested by *D. suzukii* in 2017 (β, SE, *t*, and *P* values for each comparison in text above). The different smooth regression lines represent the probability of infestation for each cultivar; the band surrounding each smooth regression line represents the 95% CIs, and the dots represent data points for each corresponding cultivar. Some dots are overlapping and incorporated into the same dot, so not all datapoints are visible.

From the 2017 comparison on the effects of Brix on infestation status between cultivars, we found there was no significant difference between Bing and Brooks (β = 0.01 ± 0.115, *t* = 0.091, *P* = 0.927). Likewise, comparisons of the effect of Brix on infestation status between Bing and Black Tartarian (β = 0.148 ± 0.081, *t* = 1.826, *P* = 0.067) and Bing and Rainier (β = 2.466 ± 1.475, *t* = 1.671, *P* = 0.094) showed no significant difference (Fig. 4).

### Firmness Assessments

**Fig. 5.**
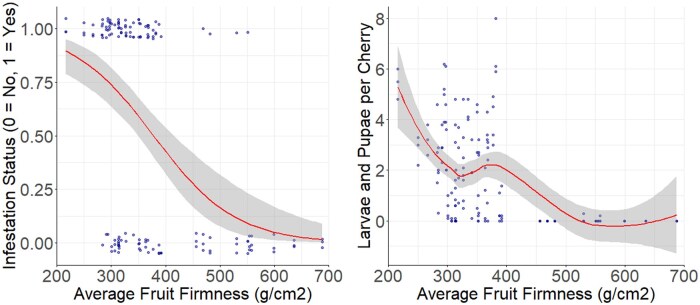
The relationship between fruit firmness and the probability of sweet cherry infestation status by *D. suzukii* (left, *P* < 0.001) and the number of larvae and pupae per cherry (right, *P* < 0.0001) in the 2013 preliminary study. In both graphs, the smooth line represents the regression line, the band surrounding the smooth line represents the 95% CIs, and dots represent data points. Some dots are overlapping and incorporated into the same dot, so not all datapoints are visible.

In the 2013 preliminary study, to examine the effects of fruit firmness on fruit infestation status, we found that firmness had a significant, negative effect on fruit infestation status (β = −0.015 ± 0.003, *t* = −4.666, *P* < 0.001, Fig. 5) as well as on the larvae and pupae per cherry (β = −0.003 ± 0.0004, *t* = −8.311, *P* < 0.001, Fig. 5). These results, as well as the results on the effect of Brix on infestation, provided justification to pursue assessments on the effects of Brix and firmness as factors affecting sweet cherry oviposition by *D. suzukii*.

**Fig. 6.**
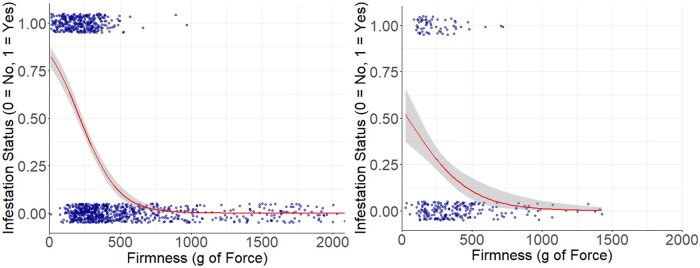
Relationship between fruit firmness and the probability of sweet cherry being infested by *D. suzukii* in (left) 2017 and (right) 2018. β, SE, *t*, and *P* values for each comparison in text above. The smooth regression line represents the probability of infestation, the band surrounding the smooth line represents the 95% CIs, and the dots represent data points. Some dots are overlapping and incorporated into the same dot, so not all datapoints are visible.

When analyzing the effects of fruit firmness on infestation status in both years, we found that fruit firmness had a significant, negative effect on fruit infestation status in 2017 (β = −0.011 ± 0.001, *t* = −9.835, *P* < 0.001, Fig. 6) and in 2018 (β = −0.013 ± 0.005, *t* = −2.64, *P* < 0.01, Fig. 6).

**Fig. 7.**
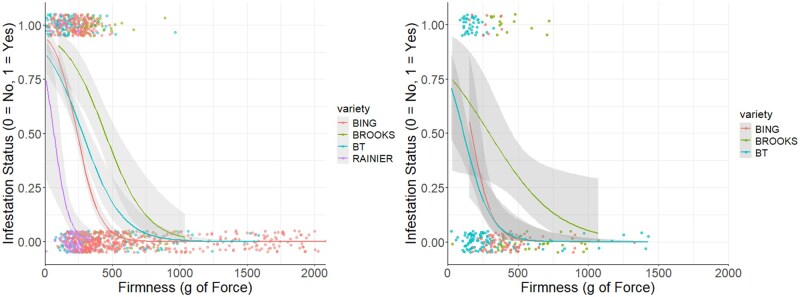
Probability of sweet cherry fruit being infested in relation to firmness for each cultivar in 2017 and 2018. Relationship between fruit firmness and the probability of each sweet cherry cultivar being infested by *D. suzukii* in 2017 ([left], β, SE, *t*, and *P* values for each comparison in text above) and 2018 ([right], the same respective values in the text below). The different smooth regression lines represent the probability of infestation for each cultivar; the band surrounding each smooth regression line represents the 95% CIs, and the dots represent data points for each corresponding cultivar. Some dots areoverlapping and incorporated into the same dot, so not all datapoints are visible.

When comparing the effects of fruit firmness on infestation status between cultivars in 2017 we found that both Brooks (β = 0.005 ± 0.001, *t* = 2.626, *P* < 0.05) and Black Tartarian (β = 0.004 ± 0.001, *t* = 2.92, *P* < 0.005) had significantly lower protective effects of firmness on infestation status than Bing while there was no significant difference in the protective effect of firmness for Rainier when compared with Bing (β = −0.006 ± 0.006, *t* = −0.992, *P* = 0.321, Fig. 7).

Due to the infestation of only one Rainier cherry during our fruit sampling, this cultivar was omitted from the 2018 analysis. When comparing the effects of fruit firmness on infestation status between cultivars in 2018 we found that the effect of firmness on infestation risk was lower for Brooks than Bing although differences were not statistically significant at *P* = 0.05 (β = 0.009 ± 0.005, *t* = 1.721, *P* = 0.085) and that there was no difference for Black Tartarian when compared with Bing (β = 0.002 ± 0.005, *t* = 0.484, *P* = 0.628, Fig. 7). It is notable that only one cherry was infested from the 2 Rainier trees sampled in 2018 even though fruit from all of the other cultivars sustained high levels of infestation. However, because the infestation was so low the Rainier fruit were omitted from the 2018 analysis. The correlation tests between sampling date and temperature for 2017 indicated that there was no difference between sampling date and average temperature (*t* = −0.4346, df = 1084, *P* = 0.6639), but in 2018 there was a negative correlation between sampling date and average temperature (*t* = −8.1713, df = 338, *P* < 0.001).

## Discussion

It is notable that the experimental design of our study is unique among the other studies cited in this discussion. Our study assesses Brix and firmness values of fruit in a natural setting over a specific time period that encompasses the ripening period for the 4 cultivars sampled in the orchard, so each cultivar will reflect changing Brix and firmness values such that fruit of other cultivars may be at different stages of maturity, as opposed to some of the laboratory-based choice and no choice assessments with specific Brix and firmness values cited here. When comparing cultivars, our infestation results should be interpreted with this difference in experimental design in mind.

Our results indicate that Brix value is positively associated with *D. suzukii* oviposition on the common sweet cherry cultivars selected for these studies, indicating a potential preference for higher Brix during host selection by adult flies, although with no differences observed between cultivars. This is in agreement with research focusing on preference of sweet cherry varieties in China that showed increased oviposition on cherries of the Hongdeng and Burlat cultivars with higher Brix (Yang et al. 2024). Further, these results support those of a laboratory study on Bing, Black Tartarian and Brooks that showed more eggs and greater developmental success on cherries of higher Brix values, although Rainier was not included in this study (Lee et al. 2011). Although susceptibility to *D. suzukii* infestation in tart cherry fruit was correlated with increased Brix values, the effect was only seen when firmness and Brix were paired together (Kamiyama and Guédot 2019). Individually, the 2 variables had no effect on fruit susceptibility. Wang et al. (2019) found that higher Brix values of 10 cherry cultivars had no effect on survival rate of larvae in controlled conditions, which contradicts the earlier study by Lee et al. (2011) on laboratory caged choice and no-choice tests on blackberries, strawberries, blueberries, cherries, and raspberries.

Results similar to our findings on the impact of Brix on *D. suzukii* oviposition were also observed on strawberries in Spain, with significantly more *D. suzukii* emerging from fruit with higher Brix values than those of lower Brix (Arnó et al. 2016), and also with grapes in Italy (Baser et al. 2018). Conversely, other research suggested that egg, larva, and adult numbers as well as development times were not impacted by Brix value (Pelton et al. 2017) and that fruits with lower Brix and pH values were preferred for oviposition (Little et al. 2017), highlighting the need for further research into this topic. Other studies that tested a variety of fruit types showed that fruit with lower Brix values resulted in significantly less *D. suzukii* oviposition and longer egg to adult development times (Lee et al. 2011, 2016, Burrack et al. 2013). The effect of Brix of the non-crop plant *Skimmia japonica* on *D. suzukii* oviposition was also assessed although no direct effect was observed, suggesting that other factors are likely responsible for oviposition preference for this shrub (Lee et al. 2015). Overall, our results support relevant research suggesting that Brix value is an important factor influencing *D. suzukii* oviposition in sweet cherries in the United States.

Our results indicate that fruit firmness significantly influenced oviposition on sweet cherries in all 3 yr of study. In 2013, lower firmness, an indicator of cherry ripening, resulted in higher greater numbers of larvae and pupae per cherry and it facilitated oviposition by females in 2017 and 2018. This effect was also reported for sweet cherries from an orchard in Beijing, China where oviposition and development of adults were inversely related to fruit firmness (Yang et al. 2024). However, in collections of ripe cherries originating from a seedling selection trial in California’s San Joaquin Valley, firmness was not found to have an effect on eggs per gram of fruit (Wang et al. 2019). An important note for consideration is that piercing of the exocarp by the adult female’s ovipositor and the subsequent larval feeding can lower fruit firmness, which may impact correlation between firmness and infestation.

Further, our studies support those reported for other fruit. For example, in North Carolina and Oregon, *D. suzukii* oviposition in blueberries increased as fruit firmness decreased (Lee et al. 2016), and Arnó et al. (2016) showed that an increase in strawberry firmness was correlated with lower numbers of *D. suzukii* larvae and pupae in Spain. In grapes, higher penetration force of exocarp was negatively correlated with egg presence in Italy (Baser et al. 2018) and Germany (Entling et al. 2019), and the same was found in Maryland raspberries (Hamby et al. 2016). In laboratory-based choice tests on blueberries, fruit with firmer exocarp experienced lower *D. suzukii* oviposition activity than those more readily susceptible to piercing (Kinjo et al. 2013). However, *D. suzukii* exhibited no preference for firmness on artificial substrates, but this is likely due to the other factors assessed (Takahara and Takahashi 2017). Here we show the importance of exocarp firmness for female *D. suzukii* during host selection in an uncaged field setting, documenting that fruit with a lower penetration force is typical for soft and stone fruit as they begin to ripen and turn color or are compromised from prior oviposition, are more susceptible to oviposition.

Other research shows that factors such as fruit integrity can play a key role during host selection and that the stage of fruit development, such as unripe, ripe, or overripe alone is not the only explanation for *D. suzukii* preference for some fruit over others (Burrack et al. 2013, Keesey et al. 2015). For example, in Georgia, grapes with pre-existing damage were found to have greater levels of oviposition than intact grapes and supported greater *D. suzukii* survival to adulthood, results that were also similar to those for intact blueberries (Grant and Sial 2016). Increased oviposition on damaged grapes was observed in laboratory no-choice tests on fruit collected from Wisconsin vineyards (Pelton et al. 2017). In a separate no-choice experiment on grapes, *D. suzukii* were found to oviposit more on and exhibit higher larval survival on previously damaged grapes than intact grapes (Ioriatti et al. 2015). Exposure of the exocarp of the fruit permits easier access for feeding and greater ease when ovipositing (Hamby et al. 2016). Fruit damage and subsequent *D. suzukii* infestation can also act as a pathway for secondary pathogens, further reducing the fruit quality (Walsh et al. 2011, Atallah et al. 2014, Baser et al. 2018). Research has confirmed this effect in California, with late-season fruit crops, even those not considered primary hosts such as grapes, supporting *D. suzukii* populations, especially when already damaged (Wang et al. 2019).

Another key factor that can influence oviposition preference is fruit color. Blueberries, blackberries, raspberries, cherries, and strawberries were found to become more susceptible to *D. suzukii* infestation as they ripen and acquire a more marketable red to purple coloration in laboratory studies by Lee et al. (2011). Preference for red coloration was also shown in grapes from Switzerland, with white grapes being less susceptible to oviposition than red grapes (Linder et al. 2014). Adult female *D. suzukii* exhibited a preference for purple over green on artificial substrates (Takahara and Takahashi 2017), as well as a preference for deep red cherries (Yang et al. 2024). In our study, ripe fruit of the only yellow-colored cultivar, Rainier, had extremely low infestation levels in both 2017 and 2018, despite its lower firmness and Brix (in 2017) than 2 of the 3 red cultivars when ripe. While the negative correlation between sampling date and average temperature observed in 2018 could have resulted in less oviposition in the later ripening Rainier cultivar, there was no significant correlation between sampling period and temperature in 2017 when a similar pattern of lower infestation of Rainier fruit relative to the other cultivars. This suggests that its yellow color may have indeed impacted our infestation results. This is supported by choice tests which showed Rainier to have the lowest average *D. suzukii* egg number of the 6 cultivars tested (Yang et al. 2024). Analysis was conducted on the effects of minimum, maximum, and average temperature in 2017 and 2018 on infestation status and no significant effects were observed, ruling out influential effects of temperature on oviposition rates. However, considering the observation that *D. suzukii* activity was present throughout fruit ripening, it is unlikely that temperature was the only other factor affecting oviposition. Limited significant differences in the relationships of Brix, firmness, and infestation between varieties highlights the role Rainier plays as an outlier. However, color may not be not the sole factor aside from Brix and firmness that influenced Rainier fruit infestation in our study. Additional research on the relationship of fruit coloration on *D. suzukii* infestation in an established orchard setting seems warranted alongside further exploration of the correlation between Brix and firmness in the fruit ripening stages. To better identify other potential factors such as *D. suzukii* abundance and pressure, trapping and monitoring throughout the season may have benefited in comparing infestation levels throughout the season with pest abundance. We recommend further field studies also focus on the egg count differences as this could provide clearer comparisons given the size and weight variations between individual fruits, as well as more trees sampled per cultivar.

The results from this study highlight the importance of Brix and firmness of these commercially important sweet cherry cultivars for *D. suzukii* host selection, and that the inter-connected web of fruit characteristics should be considered when planning Integrated Pest Management (IPM) strategies, defined as sustainable pest management using biocontrol, physical and behavioral control and cultural crop management (Asplen et al. 2015). For example, intentional breeding efforts and cultivar planting choice could be key factors in limiting *D. suzukii* pressure on farms (Burrack et al. 2013). Fruit coloration or similar factors may also play a role in cultivar planting choice depending, of course, on consumer acceptance. Fruit firmness could act as a predictor for infestation risk with less firm cultivars being selected for more frequent or more intense sampling for *D. suzukii* eggs and oviposition marks (Entling et al. 2019). Sweet cherry fruit firmness could potentially be enhanced with the application of products such as calcium silicate which has been applied to blueberries in a research study that resulted in firmer fruit exocarp and reduced oviposition compared with unsprayed blueberries (Lee et al. 2016). 

In contrast to many laboratory-based choice and no choice studies that assess factors impacting the selection of fruit for *D. suzukii* oviposition using excised and often ripe fruit, our study was conducted in a mixed cultivar sweet cherry block where fruit, sampled during the entire fruit-ripening period were naturally exposed to the *D. suzukii* adult population to determine susceptibility to infestation. We have shown that the susceptibility to and probability of sweet cherry infestation by *D. suzukii* increases as Brix value increases and exocarp firmness decreases in a field study. We highlight the importance of these 2 factors in host selection as well as cultivar differences in fruit infestation. We provide the first insight on the importance of Brix and firmness relative to *D. suzukii* oviposition in a mixed block of common commercial sweet cherry cultivars in California. We also acknowledge the importance of other factors such as damage and color when implementing IPM strategies and recommend further research on other sweet cherry cultivars and IPM techniques.

## Supplementary Material

toaf367_Supplementary_Data

## Data Availability

The datasets generated during and/or analyzed during the current study are available from the corresponding author on reasonable request.
