# Supplementary material for: Field assessment of brix and firmness affecting Drosophila suzukii (Diptera: Drosophilidae) infestation in California sweet cherry cultivars
Source: J Econ Entomol. 2026 Jan 24;119(2):1243–51. doi: 10.1093/jee/toaf367 (PMC13075821; doi:10.1093/jee/toaf367)
Supplement: toaf367_Supplementary_Data [file toaf367_supplementary_data.docx]

Supplementary Materials

GLMs assessing effects of maximum, minimum and average temperature in 2017 and 2018 on infestation status. Code and output.

Average temperature 2017

> TempInfestAve_2017 <- glm(

+ infest ~ Temp.Average + variety + Sample + firmness.g.of.force + brix,

+ data = subset(x20172018, Year == 2017),

+ family = binomial())

> summary(TempInfestAve_2017)

Call:

glm(formula = infest ~ Temp.Average + variety + Sample + firmness.g.of.force +

brix, family = binomial(), data = subset(x20172018, Year ==

2017))

Coefficients:

Estimate Std. Error z value Pr(>|z|)

(Intercept) -2.595482 1.100184 -2.359 0.0183 *

Temp.Average 0.021457 0.032200 0.666 0.5052

varietyBROOKS 1.642146 0.338755 4.848 1.25e-06 ***

varietyBT 1.362281 0.280301 4.860 1.17e-06 ***

varietyRAINIER -1.118527 0.466356 -2.398 0.0165 *

Sample -0.211596 0.019669 -10.758 < 2e-16 ***

firmness.g.of.force -0.006276 0.001010 -6.215 5.15e-10 ***

brix 0.349109 0.051984 6.716 1.87e-11 ***

---

Signif. codes: 0 ‘***’ 0.001 ‘**’ 0.01 ‘*’ 0.05 ‘.’ 0.1 ‘ ’ 1

(Dispersion parameter for binomial family taken to be 1)

Null deviance: 1348.41 on 1085 degrees of freedom

Residual deviance: 685.08 on 1078 degrees of freedom

AIC: 701.08

Number of Fisher Scoring iterations: 7

Average temperature 2018

> TempInfestAve_2018 <- glm(

+ infest ~ Temp.Average + variety + Sample + firmness.g.of.force + brix,

+ data = subset(x20172018, Year == 2018),

+ family = binomial())

> summary(TempInfestAve_2018)

Call:

glm(formula = infest ~ Temp.Average + variety + Sample + firmness.g.of.force +

brix, family = binomial(), data = subset(x20172018, Year ==

2018))

Coefficients: (1 not defined because of singularities)

Estimate Std. Error z value Pr(>|z|)

(Intercept) 1.394699 3.189367 0.437 0.6619

Temp.Average -0.034111 0.204340 -0.167 0.8674

varietyBROOKS 2.395566 0.589417 4.064 4.82e-05 ***

varietyBT 0.224365 0.500602 0.448 0.6540

Sample -0.075738 0.041221 -1.837 0.0662 .

firmness.g.of.force -0.007164 0.001723 -4.158 3.21e-05 ***

brix NA NA NA NA

---

Signif. codes: 0 ‘***’ 0.001 ‘**’ 0.01 ‘*’ 0.05 ‘.’ 0.1 ‘ ’ 1

(Dispersion parameter for binomial family taken to be 1)

Null deviance: 288.01 on 268 degrees of freedom

Residual deviance: 237.43 on 263 degrees of freedom

AIC: 249.43

Number of Fisher Scoring iterations: 6

Maximum temperature 2017

> TempInfestMax_2017 <- glm(

+ infest ~ Temp.Max + variety + Sample + firmness.g.of.force + brix,

+ data = subset(x20172018, Year == 2017),

+ family = binomial())

> summary(TempInfestMax_2017)

Call:

glm(formula = infest ~ Temp.Max + variety + Sample + firmness.g.of.force +

brix, family = binomial(), data = subset(x20172018, Year ==

2017))

Coefficients:

Estimate Std. Error z value Pr(>|z|)

(Intercept) -2.305049 1.108388 -2.080 0.0376 *

Temp.Max 0.001705 0.021686 0.079 0.9373

varietyBROOKS 1.652969 0.339194 4.873 1.10e-06 ***

varietyBT 1.379389 0.279295 4.939 7.86e-07 ***

varietyRAINIER -1.086785 0.464272 -2.341 0.0192 *

Sample -0.211787 0.019680 -10.762 < 2e-16 ***

firmness.g.of.force -0.006226 0.001006 -6.191 5.98e-10 ***

brix 0.353462 0.051776 6.827 8.69e-12 ***

---

Signif. codes: 0 ‘***’ 0.001 ‘**’ 0.01 ‘*’ 0.05 ‘.’ 0.1 ‘ ’ 1

(Dispersion parameter for binomial family taken to be 1)

Null deviance: 1348.41 on 1085 degrees of freedom

Residual deviance: 685.51 on 1078 degrees of freedom

AIC: 701.51

Number of Fisher Scoring iterations: 7

Maximum temperature 2018

> TempInfestMax_2018 <- glm(

+ infest ~ Temp.Max + variety + Sample + firmness.g.of.force + brix,

+ data = subset(x20172018, Year == 2018),

+ family = binomial())

> summary(TempInfestMax_2018)

Call:

glm(formula = infest ~ Temp.Max + variety + Sample + firmness.g.of.force +

brix, family = binomial(), data = subset(x20172018, Year ==

2018))

Coefficients: (1 not defined because of singularities)

Estimate Std. Error z value Pr(>|z|)

(Intercept) 0.653316 1.602668 0.408 0.683536

Temp.Max 0.010935 0.070865 0.154 0.877371

varietyBROOKS 2.332953 0.639907 3.646 0.000267 ***

varietyBT 0.178119 0.528763 0.337 0.736223

Sample -0.076043 0.041222 -1.845 0.065078 .

firmness.g.of.force -0.007309 0.001819 -4.018 5.87e-05 ***

brix NA NA NA NA

---

Signif. codes: 0 ‘***’ 0.001 ‘**’ 0.01 ‘*’ 0.05 ‘.’ 0.1 ‘ ’ 1

(Dispersion parameter for binomial family taken to be 1)

Null deviance: 288.01 on 268 degrees of freedom

Residual deviance: 237.44 on 263 degrees of freedom

AIC: 249.44

Number of Fisher Scoring iterations: 6

Minimum temperature 2017

> TempInfestMin_2017 <- glm(

+ infest ~ Temp.Min + variety + Sample + firmness.g.of.force + brix,

+ data = subset(x20172018, Year == 2017),

+ family = binomial())

> summary(TempInfestMin_2017)

Call:

glm(formula = infest ~ Temp.Min + variety + Sample + firmness.g.of.force +

brix, family = binomial(), data = subset(x20172018, Year ==

2017))

Coefficients:

Estimate Std. Error z value Pr(>|z|)

(Intercept) -2.944207 1.049208 -2.806 0.00501 **

Temp.Min 0.097972 0.050527 1.939 0.05250 .

varietyBROOKS 1.595323 0.337193 4.731 2.23e-06 ***

varietyBT 1.268694 0.284397 4.461 8.16e-06 ***

varietyRAINIER -1.203896 0.470946 -2.556 0.01058 *

Sample -0.210781 0.019653 -10.725 < 2e-16 ***

firmness.g.of.force -0.006730 0.001052 -6.397 1.59e-10 ***

brix 0.336948 0.052374 6.434 1.25e-10 ***

---

Signif. codes: 0 ‘***’ 0.001 ‘**’ 0.01 ‘*’ 0.05 ‘.’ 0.1 ‘ ’ 1

(Dispersion parameter for binomial family taken to be 1)

Null deviance: 1348.41 on 1085 degrees of freedom

Residual deviance: 681.75 on 1078 degrees of freedom

AIC: 697.75

Number of Fisher Scoring iterations: 7

Minimum temperature 2018

> TempInfestMin_2018 <- glm(

+ infest ~ Temp.Min + variety + Sample + firmness.g.of.force + brix,

+ data = subset(x20172018, Year == 2018),

+ family = binomial())

> summary(TempInfestMin_2018)

Call:

glm(formula = infest ~ Temp.Min + variety + Sample + firmness.g.of.force +

brix, family = binomial(), data = subset(x20172018, Year ==

2018))

Coefficients: (1 not defined because of singularities)

Estimate Std. Error z value Pr(>|z|)

(Intercept) -0.443312 2.130276 -0.208 0.835150

Temp.Min 0.129179 0.197831 0.653 0.513771

varietyBROOKS 2.290332 0.590936 3.876 0.000106 ***

varietyBT 0.202895 0.490210 0.414 0.678952

Sample -0.076816 0.041290 -1.860 0.062831 .

firmness.g.of.force -0.007185 0.001711 -4.199 2.68e-05 ***

brix NA NA NA NA

---

Signif. codes: 0 ‘***’ 0.001 ‘**’ 0.01 ‘*’ 0.05 ‘.’ 0.1 ‘ ’ 1

(Dispersion parameter for binomial family taken to be 1)

Null deviance: 288.01 on 268 degrees of freedom

Residual deviance: 237.03 on 263 degrees of freedom

AIC: 249.03

Number of Fisher Scoring iterations: 6

| **Tree ID** | **Sample number** | **Larvae number** | **Pupae number** |
| --- | --- | --- | --- |
| 1L | 318 | 222 | 156 |
| 1N | 327 | 387 | 245 |
| 2D | 296 | 328 | 305 |
| 2K | 243 | 238 | 239 |
| 2M | 239 | 285 | 153 |

**Table 1.** Fruit sample number, larvae and pupae numbers for each tree type sampled in 2013.
